# The effect of cochlear implant surgery on vestibular function in adults: A meta-analysis study

**DOI:** 10.3389/fneur.2022.947589

**Published:** 2022-08-10

**Authors:** Fabiane de Castro Vaz, Leonardo Petrus, Wagner Rodrigues Martins, Isabella Monteiro de Castro Silva, Jade Arielly Oliveira Lima, Nycolle Margarida da Silva Santos, Natália Turri-Silva, Fayez Bahmad

**Affiliations:** ^1^Postgraduate Program in Health Sciences at the Faculty of Health Sciences, University of Brasília, Brasília, DF, Brazil; ^2^3ID Ensino, 3 ID Prevenção e Reabilitação Geriátrica, Brasília, DF, Brazil; ^3^Health and Technologies in Health Sciences Program, University of Brasília, Brasília, DF, Brazil; ^4^College of Physical Therapy, University of Brasília, Brasília, DF, Brazil; ^5^College of Physical Education, University of Brasília, Brasília, DF, Brazil; ^6^Faculty of Speech Therapy, University of Brasília, Brasília, DF, Brazil

**Keywords:** vestibular loss, vestibular function, cochlear implant, balance, vertigo, dizziness

## Abstract

**Issue:**

The findings in literature indicate inconsistency in the complications caused by the implant of electrodes in the cochlea; vestibular alterations and balance disorders are mentioned as the most likely.

**Purpose:**

To evaluate, in literature, through the results of multiple vestibular function tests, the effects of cochlear implant surgery on postural stability in adult patients and to analyze.

**Hypothesis:**

From the PICO strategy, where the Population focuses on adults, Intervention is cochlear implant surgery, Comparisons are between implanted patients, and Outcomes are the results of the assessment of cochlear function, the research question was formulated: Are there deficits in vestibular function in adults undergoing cochlear implant placement?

**Method:**

Systematic review based on cohort, case–control, and cross-sectional observational studies. Information sources: Databases between 1980 and 2021, namely, PubMed, Cinahl, Web Of Science, Cochrane, and Scopus. Search strategy using Mesh terms: “Adult,” “Cochlear Implant,” “Postural Balance,” “Posturography,” “Cochlear Implant,” “Dizziness,” “Vertigo,” “Vestibular Functional Tests,”and “Caloric Tests.” Populational inclusion criteria: studies with adult patients; intervention: cochlear implant placement surgery; comparison: analysis of a vestibular function with vestibular test results and pre- and postoperative symptoms; outcome: studies with at least one of the vestibular function tests, such as computerized vectoelectronystagmography (VENG), vestibular-evoked myogenic potentials (VEMPs), caloric test, video head impulse test (VHIT), head impulse test (HIT), videonystagmography, (VNG) and static and dynamic posturography. Exclusion criteria: studies without records of pre- and postoperative data collection and studies with populations under 18 years of age. Screening based on the reading of abstracts and titles was performed independently by two reviewers. In the end, with the intermediation of a third reviewer, manuscripts were included. Risk of bias analysis, performed by two other authors, occurred using the JBI “Critical Appraisal Checklist.”

**Results:**

Of the 757 studies, 38 articles met the inclusion criteria. VEMP was the most commonly used test by the studies (44.7%), followed by the caloric test (36.8%) and vHIT (23.6%). Most studies performed more than one test to assess vestibular function.

**Conclusion:**

Among all vestibular tests investigated, the deleterious effects on vestibular function after cochlear implant surgery were detected with statistical significance (*P* < 0.05) using VEMP and caloric test. Comparing abnormal and normal results after implant surgery, the vestibular apparatus was evaluated as having abnormal results after cochlear implant surgery only in the VEMP test. The other tests analyzed maintained a percentage mostly considered normal results.

**Systematic review registration:**

identifier: CRD42020198872.

## Introduction

As one of the main systems essential for maintaining balance, the vestibular system is responsible for ensuring postural control, gaze stabilization, and spatial orientation (1).

Currently, several tests are available to assess the most diverse aspects of the condition of the vestibular apparatus. For the symptoms presented in the post-surgical period, it is essential to investigate the deficiency of different structures within the vestibular system, which can be evaluated by exams such as videonystagmography (VNG), vestibular evoked myogenic potential (VEMPs), test analysis calories, video head impulse test (VHIT), head impulse test (HIT), and static and dynamic posturography.

The VEMP test assesses saccular and inferior vestibular nerve function, indirectly measuring vestibular function through the vestibulo-colic reflex that depends on the integrity of the saccular macula, inferior vestibular nerve, vestibular nuclei, vestibulospinal pathways, and the muscle sternocleidomastoid. They are evoked by loud acoustic stimuli in the ipsilateral ear and recorded using surface electrodes over the sternocleidomastoid muscle (2, 3).

The vHIT records and measures the speed of eye and head movements, and being a more complete version of the HIT that uses only visual observation, both use the observation of VOR for the diagnosis of vestibular alteration. Patients with alteration present corrective eye movement (saccades) during or after head impulse (4, 5). Caloric testing is considered a standard test to assess the vestibulo-ocular reflex (VOR) by stimulating the lateral semicircular canals at low frequencies using water or air. However, this form of stimulation is considered invasive and requires microscopic examination of the ear before performing the test. As a result, the HIT and vHIT tests are being increasingly used as they non-invasively stimulate the horizontal and vertical semicircular canals with high frequencies (6). Even so, what should move the indication of the test is its benefit in the elaboration of the final diagnosis.

How well you are using your visual, vestibular, and proprioceptive systems to stay balanced can be determined through Computerized Dynamic Posturography (PDC), an exam used in the quantitative assessment of body balance. The PDC clearly and consistently demonstrates the gradual evolution of the postural performance and body balance of each patient throughout all assessments, and, for example, finding better postural performance after HF by means of PDC in the long term (7, 8).

The occurrence of complaints of dizziness in the postoperative period of patients undergoing cochlear implant (CI) surgery has been reported in the literature for decades.

Although many articles on the vestibular system and its correlation with the cochlear implant have been carried out, the mechanisms of CI interference on vestibular function and after the procedure are still not fully understood. Some possible explanations would be the process during the surgery, with the placement of the implant in a traumatic way; or some degree of destruction generated in the vestibular receptors due to the loss and pressure changes of the endolymph or perilymph; or even change in bone formation and the membranous labyrinth of endolymph and perilymph due to inflammation, scar formation, saccular membrane distortion, fibrosis, foreign body reaction, changes in lymph fluid composition, and self-regenerative abilities of receptors (9).

Studies on these issues are divergent in the literature. Due to the different opinions and studies, authors disagree with their findings. Between authors who describe that CI can improve body balance (8, 10–12), those who believe that CI negatively interferes with vestibular function (9, 13–19), and authors who maintain that CI has no effect in this regard (16, 20–23), there are many different studies and evaluation methodologies.

This study aims to evaluate the effects of cochlear implant surgery on balance in adults and to verify, through the analysis of vestibular exam results, whether patients undergoing this procedure had deficits in the vestibular system. Therefore, the central question of this meta-analysis, developed by the PICO strategy (Population, Interventions, Comparisons, Outcomes), aims, through quantitative data and clinical measures of vestibular function assessment tests, to demonstrate whether there is a change in the vestibular system after surgery cochlear implant in adults.

## Materials and methods

This meta-analysis was written following the Methodological Expectations of Cochrane Intervention Reviews (MECIR) and the PRISMA checklist criteria, and its protocol was registered on 14/08/2020 in the international prospective registry of systematic reviews-PROSPERO, under the registration number CRD42020198872.

### Eligibility criteria

Observational studies published between 1980 and 2021 in English, Portuguese, and Spanish languages were investigated. Titles and abstracts were reviewed by two independent reviewers.

The included studies had to meet the eligibility criteria structured according to the PICO strategy:

(1) Population: studies with patients older than 18 years; (2) intervention: cochlear implant placement surgery; (3) comparison: analysis of vestibular function in pre- and post-operative implanted patients, or, studies that analyzed vestibular function comparing implanted and non-implanted patients (control group); and (4) outcome: studies including at least one of the following vestibular function tests: (1) vectoelectronystagmography (VENG); (2) vestibular evoked myogenic potential (VEMPs); (3) caloric test; (4) video head impulse test (VHIT); (5) head impulse test (HIT); (6) videonystagmography (VNG); and (7) static posturography and dynamic posturography.

Studies that did not record pre-and postoperative data collection or had samples with participants under 18 years of age were excluded from the systematic review.

Studies that reported numbers of normal and abnormal patients for the following tests: clinical head impulse test (HIT), caloric, and vestibular evoked myogenic potential (VEMP) testing were included. Studies that reported raw or average data and standard deviations for posturography [Sensory Organization Test (SOT) conditions 5 and 6] were also included.

We have included in the meta-analysis only studies that have reported the number of subjects with normal pre-and postoperative results.

### Information source/search

The literature review was performed on 04/12/2021 using the following databases: (1) PubMed, (2) Cinahl, (3) Web Of Science, (4) Cochrane, and (5) Scopus. The following search strategy was developed using a combination of Mesh (Medical Subject Headings) terms and other descriptors:

#1 (Adult or Young Adult) and (Dizziness Vertigo).

#2 (“Cochlear Implants” OR “Cochlear implant”).

#3 “Postural Balance” OR “Postural Control” OR Gait OR Mobility OR Balance OR “Standing Balance“ OR Walking OR “Body-Sway” OR “Physical Functional Performance” OR “Functional Performance Tests” OR “Balance Performance” OR “Clinical Balance Measures” OR Posturography OR “Dynamic Posturography” OR “STATIC Posturography” OR “force plate” OR statokinesigram OR “center of pressure” OR “center of pressure velocity” OR VEMP OR “Vestibular Evoked Myogenic Potentials” OR VENG OR vectoelectronystagmography OR VNG OR videonystagmography OR “Vestibular Function Tests” OR “Caloric Tests” OR HIT OR “Head Impulse Test” OR “Vídeo Head Impulse Test” OR VHIT.

### Study selection/data collection process/data list

After identifying the articles for analysis, a complete reading of the studies was independently performed by two reviewers.

For each study, the following were extracted: author's name, year of publication, type of study, comparison, sample size, age, age (mean and standard deviation), tests applied, type of surgery, postoperative symptoms, implant side, implanted ear, causes of deafness, and vestibular test results (Supplementary Table 1).

### Risk of bias in each study

Studies that met the inclusion criteria have been included for this step, and each study type was analyzed according to the Joanna Briggs Institute risk of bias assessment using the JBI “Critical Appraisal Checklist” for Case-Control, cohort, and cross-sectional analytic studies. This analysis was performed by two judges independently, if necessary, a third one was requested for a final opinion.

### Data presentation

Different tests exist to evaluate different aspects of the state of the vestibular apparatus. The HIT is one test that assesses the vestibulo-ocular function. Other tests objectively evaluate parameters associated with different parts of the vestibular apparatus; however, they do not measure the function of the vestibular system. Such tests include the caloric and VEMP tests.

Posturography is a set of tests that assess the integrative vestibular performance associated with the maintenance of posture, where the vestibular function integrates with other sensory inputs (such as vision and proprioception to maintain posture). When applying the SOT test, posturography assesses the state of compensation because all the movements are sway-referenced, with no induced movements.

### Summary measures

Five separate meta-analyses were conducted, one for each test. For HIT, VHIT, VNG, caloric, and VEMP tests, the outcome was obtained from the proportion of subjects with normal test results before and after surgery, and effect size was measured using the log of relative risk (RR) because the results are reported in a dichotomous manner (i.e., normal or hypo/arreflexia). Meta-analyses were conducted using the RStudio software version 2022.02.1+461 using meta packages.

The reference values that were described in the studies for normal results are shown in Table 1. The results identified as normal for each test in the studies differ from each other; however, they were grouped in the table as the studies were similar in their cut-off points (Table 1).

**Table 1:** Cut-off points used to discriminate reference values for tests considered normal.

**Reference**	**Caloric test**	**Reference**	**cVEMP oVEMP**	**Reference**	**Hit**	**Reference**	**VHIT**	**Reference**	**VNG**
Batuecas-Caletrio et al. (24)	From the Jongkees formula, sides differences >25% were considered altered. Differences >10% between postoperative response and preoperative tests were considered abnormal.	Colin et al. (25), Barbara et al. (26), Coordes et al. (9), Imai et al. (27), Louza et al. (28), Nordfalk et al. (29), Nordfalk et al. (30), Rasmussen et al. (31)	Does not describe potential latency and amplitude	Colin et al. (25), Meli et al. (32), Melvin et al. (33)	Qualitative description of ocular fixation	Maheu et al. (34)	NI	Batuecas-Caletrio et al. (24), Nordfalk et al. (30)	From the Jongkees formula, sides differences >25% were considered altered. Differences >10% between postoperative response and preoperative tests were considered abnormal.
Guan et al. (21), Piker et al. (35), Rasmussen et al. (31), West et al. (36)	From the Jongkees formula, sides differences >25% were considered altered.	Ernst et al. (37)	NI	Jutila et al. (38)	Gain <0.84 or asymmetry in gain >10%	Bittar et al. (39), Piker et al. (35), Batuecas-Caletrio et al. (24)	Gain > 0.8	Filipo et al. (40)*	In caloric testing, side preponderance (SP) from the Jongkees formula, an inter-ear difference >20%. *This study presented only the cutoff point used for normal results in the caloric test, the other values of the tests used in the VNG in this study were not discriminated
Brey et al. (15), Filipo et al. (40), Kluenter et al. (41), Parietti-Winkler et al. (42)	From the Jongkees formula, an inter-ear difference >20%	Guan et al. (43)	Amplitude asymmetry ratio (AR): (right amplitude-left amplitude) | /(right + left amplitude) × 100%. Abnormal AR > 0.34 or no repeatable waveform	Migliaccio et al. (21)	Gain <0.74 for horizontal canals and <0.64 for vertical canals	Rasmussen et al. (31), West et al. (36)	Gain > 0,7	Colin et al. (25), Nordfalk et al. (29)	NI
Miwa et al. (44), Melvin et al. (33)	The maximum slow-phase velocity of <10 o/s was considered to represent hypofunction	Melvin et al. (33)	Reduced saccular function by CI-VEMP present preoperatively and absent postoperatively or with an increase in threshold > 10 dB postoperatively						
Ito (45)	The maximum slow-phase velocity of <7o/s was considered to represent hypofunction	Tsukada and Usami (46)	Amplitude asymmetry ratio (AR) = (amplitude of CI side–amplitude of non-CI side)*100/(amplitude of CI side + amplitude of non-CI side). Decreased reaction on the CI side <30%; decreased reaction on the non-CI side >30% or no reaction when amplitude bilaterally absent.						
Black et al. (47), Kiyomizu et al. (48), Vibert et al. (16), Nordfalk et al. (29), Nordfalk et al. (30)	NI	West et al. (36)	Difference between primarily binary (response) and secondarily amplitude size > 20 mV was abnormal						

For posturography, the outcome measure should be the average difference of the scores and the effect size measured using the average difference of the scores before and after surgery, so that the random-effects model would be used because of the expected variability in testing conditions and interpretation of results at different testing centers; however, due to the low number of studies with these available data, it was not possible to perform a meta-analysis of the data from these tests because those presented by the studies are incomparable. For posturography, only two studies (15, 41) reported the average and standard deviation of the same sensory condition.

Posturography data or other analyses of signs and symptoms could not be included in the meta-analysis, and a qualitative analysis was carried out for these studies.

## Results

**Figure 1:**
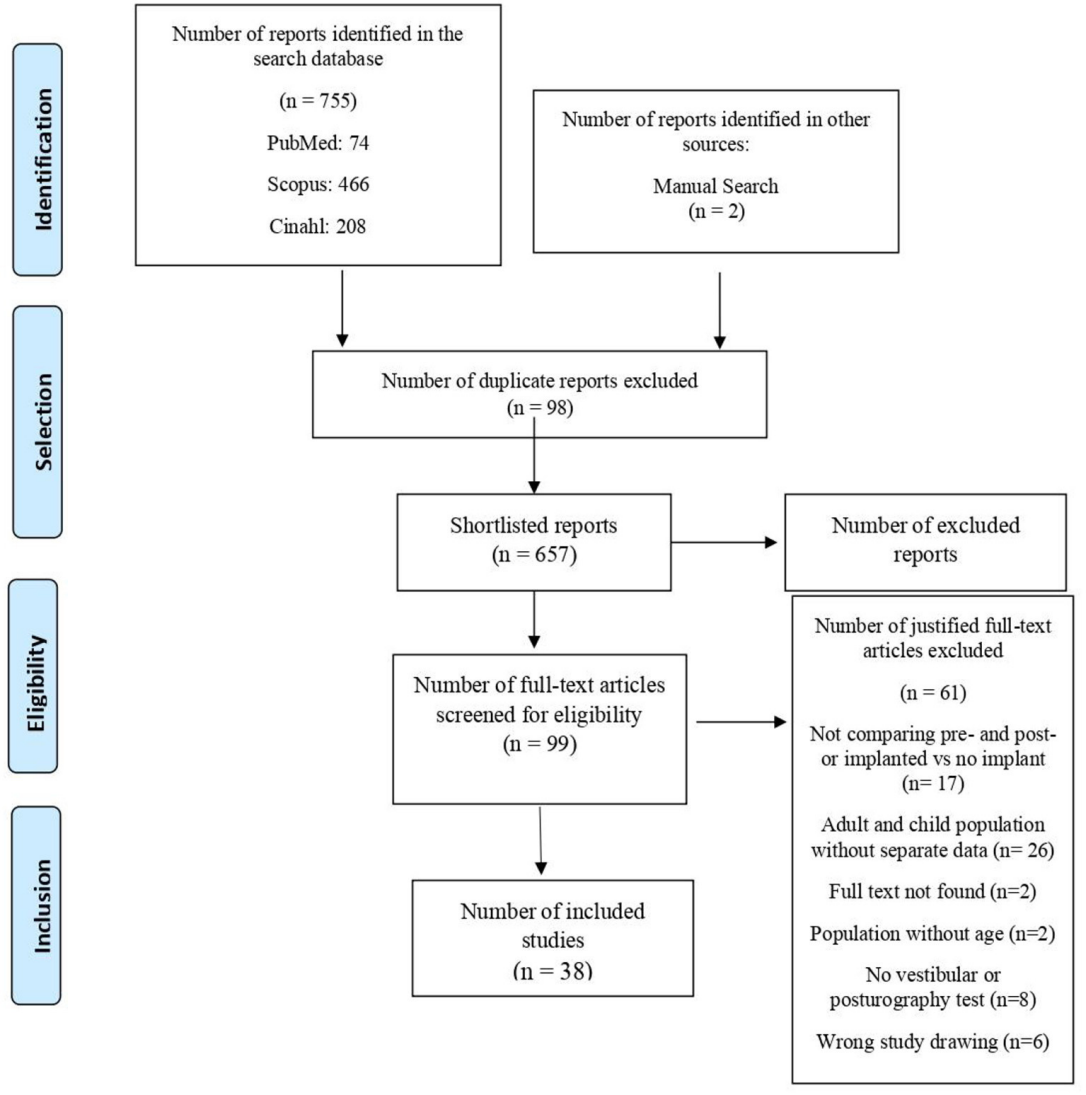
Diagram of search and study selection process adapted from PRISMA.

Of the 757 studies found, 558 articles were excluded from titles and abstracts for not meeting the eligibility criteria, and, 98 for reasons of duplicates (Figure 1). Then, we excluded 61 articles after reading the full text of each of the 99 selected publications.

The remaining 38 studies that met all criteria were included and described separately. The summary of the general characteristics of the studies included in the systematic review, such as type of surgery performed, uni or bilateral implantation, and studies that included subjective measures, such as dizziness or imbalance before and after implantation and their descriptions, are detailed in (Supplementary Table 1).

Regarding the general characteristics of the articles included in the systematic review, 6 articles carried out their research using a control group compared to those implanted (23, 34, 41, 42, 49), while the remaining 32 studies compared the results of their patients before and after cochlear implant (Supplementary Table 1).

Of the included studies, 2 analyzed patients with unilateral, bilateral, and second implants; 19 studies analyzed patients with unilateral implants; 5 studies contained samples with bilateral and unilateral; however, 12 did not mention in their studies whether the surgery was unilateral or bilateral (Supplementary Table 1).

In the analyzed articles, the most used test in the studies was the VEMP (44.7%), followed by the caloric test (36.8%) and vHIT (23.6%). In addition to these, VNG (21.05%) and HIT were also performed which evaluates the VOR used in 13.1% of the studies. Posturography was also performed in 28.9% of the articles. Most studies performed more than one test to assess vestibular function and balance (Supplementary Table 1).

Among all the 38 articles, after judgment based on the risk of bias analysis (JBI), analytic cross-sectional studies, and according to each study type, 25 studies showed a low risk of bias (9, 14, 16, 24, 27, 31, 33, 35, 36, 38, 39, 42–44, 46, 48–55), 10 articles (15, 23, 25, 26, 28–30, 32, 34, 47) showed a moderate risk of bias, and 3 articles (21, 40, 45) had a high risk of bias.

For the meta-analysis, we only consider 30 of these articles, which include the data from pre-and post-operative tests. The remaining 8 articles were not included in the meta-analysis because they did not have the pre-and postoperative data.

### Qualitative analysis

Regarding reports of pre-existing symptoms, the majority of articles did not mention them, however, about post symptoms, 17 cited vertigo as the main symptom (9, 14–16, 21, 25, 26, 28, 30, 33, 39, 40, 44, 48, 50–52), and in 5 of these (25, 26, 28, 30, 39, 51), the round window was the surgical intervention, 5 (16, 21, 33, 40, 44) had cochleostomy, in 1 (25) both interventions were present, and the others did not mention the surgery, despite having reported the symptoms. Of all the articles eligible for this review, 44.7% (9, 14–16, 21, 25, 26, 28, 30, 33, 39, 40, 44, 48, 50–52) reported vertigo as a post-implantation symptom, followed by dizziness and imbalance (16, 29, 32, 35, 36, 38, 39, 44, 45, 48, 50, 51, 53); however, in 7.8% (34, 54, 55) of the studies, there were no reports or citations of any referred symptom (Supplementary Table 1).

Of the articles that reported vertigo as the main symptom, most of these studies (15, 16, 21, 33, 39, 44, 48, 50–52) reported only the appearance of postoperative vertigo and did not provide data on whether there were subjective symptoms in these patients before surgery. Five studies (9, 25, 26, 28, 40) presented patients who did not report preoperative symptoms but presented postoperative symptoms (Supplementary Table 1).

The results of the studies that investigated posturography, especially in conditions 5 and 6, are incomparable; therefore, a meta-analysis could not be conducted, since only two studies presented data with mean and standard deviation before and after surgery (15, 41). Brey et al. found a non-significant difference between pre- and post-implantation, where the difference in scores for conditions 5 and 6 was very subtle. Overall, the performance of postural stability did not appear to be affected by CI surgery (15). Stieger's analysis (55) indicated that 5 of 15 (33%) patients who had normal balance preoperatively had pathological balance control postoperatively. Furthermore, 3 of the 12 (58%) patients who were <60 years of age had pathological balance postoperatively.

### VEMP test results

**Figure 2:**
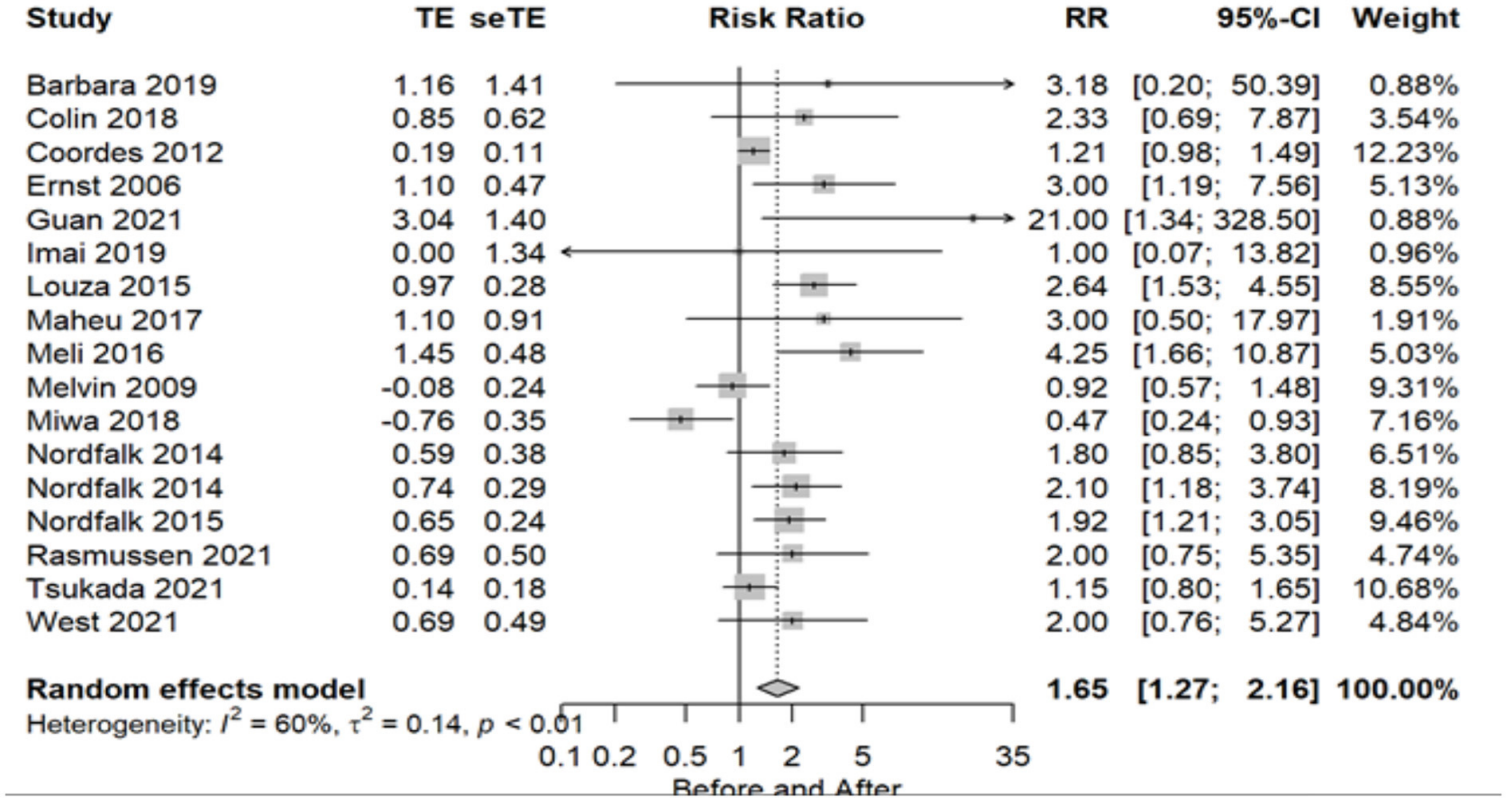
Forest plot (showing relative effect sizes) for the VEMP test.

A total of 17 (56.6%) studies was included in the meta-analysis of the VEMP, with 223 subjects as normal before and 123 as normal after implantation. All included studies used cVEMP. Statistical analysis revealed a significant detrimental effect of CI surgery on VEMP test results (RR = 1.65 95% CI = 1.27–2.16 *P* = 0.0002). There was substantial heterogeneity across studies (I2 = 60%, *P* = 0.01). The forest plot indicating the relative strength of each study included in the meta-analysis is illustrated in Figure 2. The table containing all the articles that used the VEMP test for their analysis is found in Table 2.

**Table 2:** Number of subjects with normal and abnormal testing results before and after surgery in studies included in the meta-analysis for the VEMP test.

**Study**	**Normal Pre**	**Abnormal pre**	**Normal post**	**Abnormal post**	**Number pre**	**Number post**
Colin et al. (25)	7	15	3	19	22	22
Miwa et al. (44)	4	5	1	8	9	9
Ernst et al. (37)	12	6	4	14	18	18
Imai et al. (27)	1	7	1	7	8	8
Louza et al. (28)	29	12	11	30	41	41
Maheu et al. (34)	3	1	1	3	4	4
Meli et al. (32)	17	8	4	21	25	25
Melvin et al. (33)	12	7	11	5	19	16
Nordfalk et al. (29)	9	3	5	7	12	12
Nordfalk et al. (29)	RW:12 CCL:9	RW:4 CCL:8	RW:5 CCL:5	RW:11 CCL:12	RW:16 CCL:17	RW:16 CCL:17
Nordfalk et al. (30)	25	8	13	20	33	33
Barbara et al. (26)	5	17	0	6	22	6
Coordes et al. (9)	27	0	14	3	17	117
Rasmussen et al. (31)	10	33	5	38	43	43
Tsukada and Usami (46)	31	25	27	29	56	56
West et al. (36)	10	26	5	31	36	36

### Caloric test results

**Figure 3:**
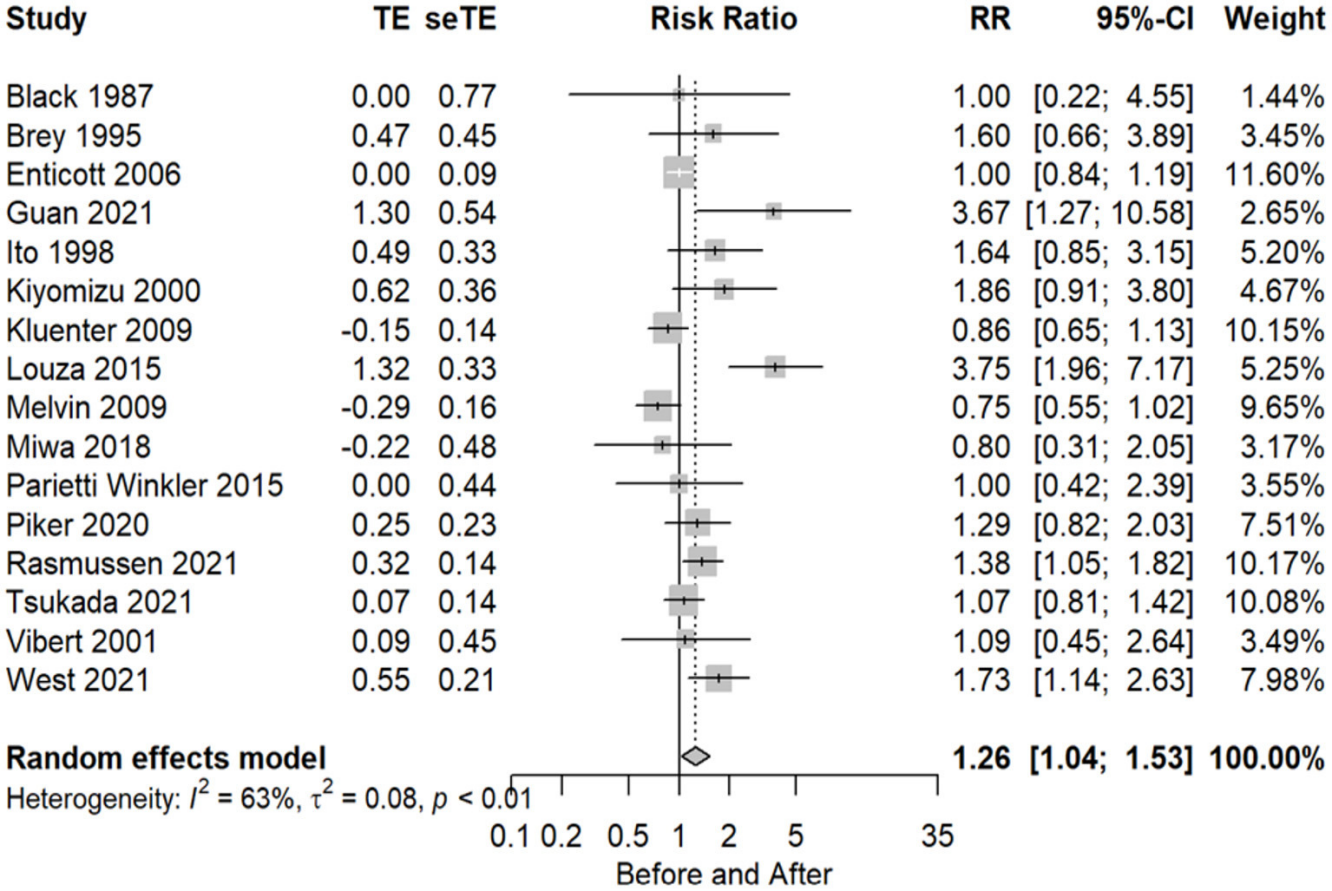
Forest plot (showing relative effect sizes) for the caloric test.

The number of subjects with normal and abnormal test results before and after CI surgery included in the meta-analysis of the caloric test was 303 as normal before and 203 after the implant, with a total of 16 articles (53.3%) using this test. Statistical analysis revealed a significant effect of CI surgery on caloric test results (RR = 1.26 95% CI = 1.04–1.53, *P* = 0.0197). There was considerable heterogeneity observed across studies (I2 = 63%, *P* = 0.01). The forest plot indicating the relative strength of each study included in the meta-analysis is illustrated in Figure 3. The table containing all the articles that used the Caloric test for their analysis is found in Table 3.

**Table 3:** Number of subjects with normal and abnormal testing results before and after surgery in studies included in the meta-analysis for the caloric test.

**Study**	**Normal pre**	**Abnormal pre**	**Normal post**	**Abnormal post**	**Number pre**	**Number post**
Miwa, et al. (44)	4	5	5	4	9	9
Vibert et al. (16)	6	5	4	4	11	8
Brey et al. (15)	8	9	5	12	17	17
Parietti-Winkler et al. (42)	5	5	5	5	10	10
Black et al. (47)	2	3	2	3	5	5
Enticott et al. (14)	65	21	65	21	86	86
Kiyomizu et al. (48)	13	10	7	16	23	23
Kluenter et al. (41)	18	6	21	3	24	24
Louza et al. (28)	30	11	8	33	41	41
Melvin et al. (33)	14	6	15	1	20	16
Piker et al. (35)	9	1	7	3	10	10
Rasmussen et al. (31)	36	7	26	17	43	43
Tsukada and Usami (46)	38	18	31	18	56	49
Westl et al. (36)	26	8	15	19	34	34
Guan et al. (43)	11	4	3	12	15	15
Ito et al. (45)	18	37	11	44	55	55

### HIT test results

**Figure 4:**
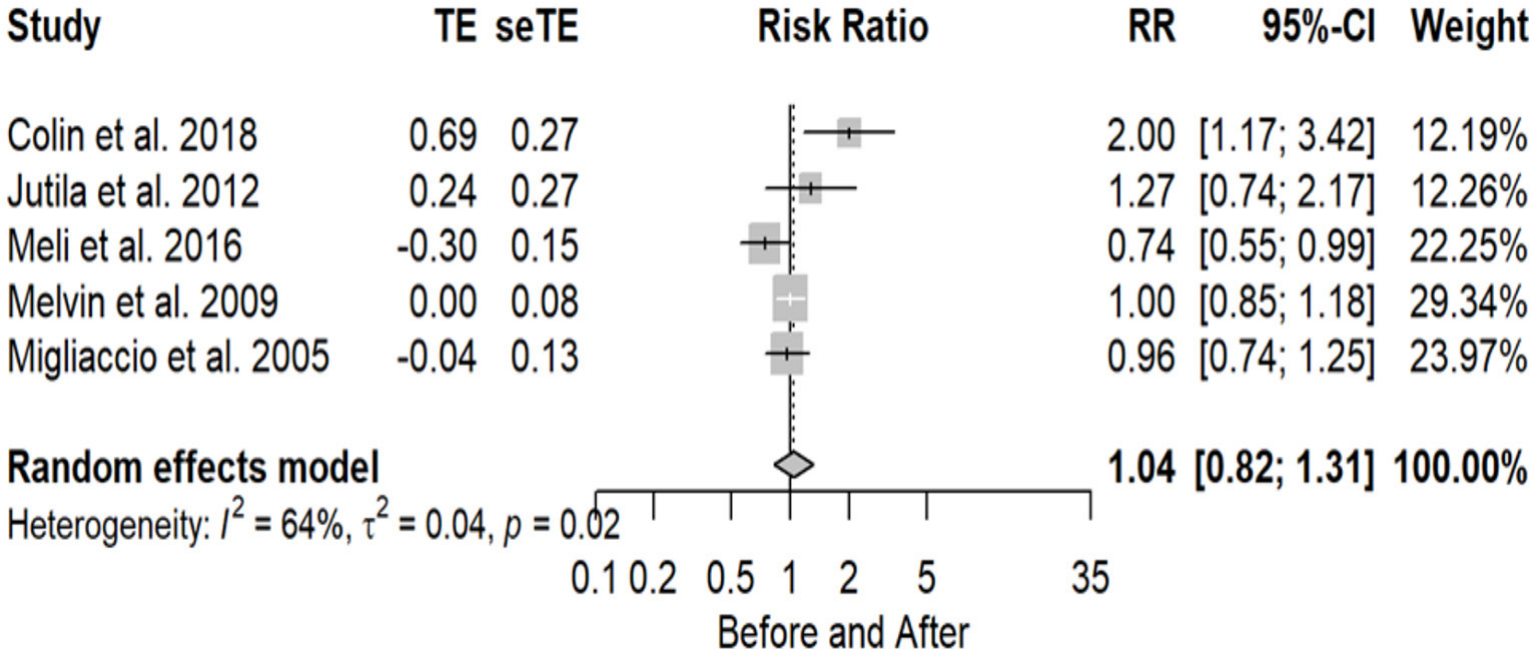
Forest plot (showing relative effect sizes) for the HIT test.

The number of implant patients included in the meta-analysis, evaluated using the HIT test, with normal and abnormal test results before and after CI surgery totaled 5 studies (16.6%), with 82 subjects as normal before and 67 after surgery. Statistical analysis revealed a non-significant effect of CI surgery on HIT test results (RR = 1.04 95% CI = 0.82–1.31 *P* = 0.7563). HIT proved to be an inconclusive test, with no significant evidence of substantial variability in the results observed in these studies (I2 = 64%, *P* = 0.02). The forest plot indicating the relative strength of each study included in the meta-analysis is illustrated in Figure 4. The table containing all the articles that used the HIT test for their analysis is found in Table 4.

**Table 4:** Number of subjects with normal and abnormal testing results before and after surgery in studies included in the meta-analysis for HIT test.

**Study**	**Normal pre**	**Abnormal pre**	**Normal post**	**Abnormal post**	**Number pre**	**Number post**
Colin et al. (25)	18	4	9	13	22	22
Jutila et al. (53)	19	25	15	29	44	44
Meli et al. (32)	17	8	23	2	25	25
Migliaccio et al. (21)	14	2	10	1	16	11
Melvin et al. (33)	14	0	10	0	14	10

### vHIT test results

**Figure 5:**
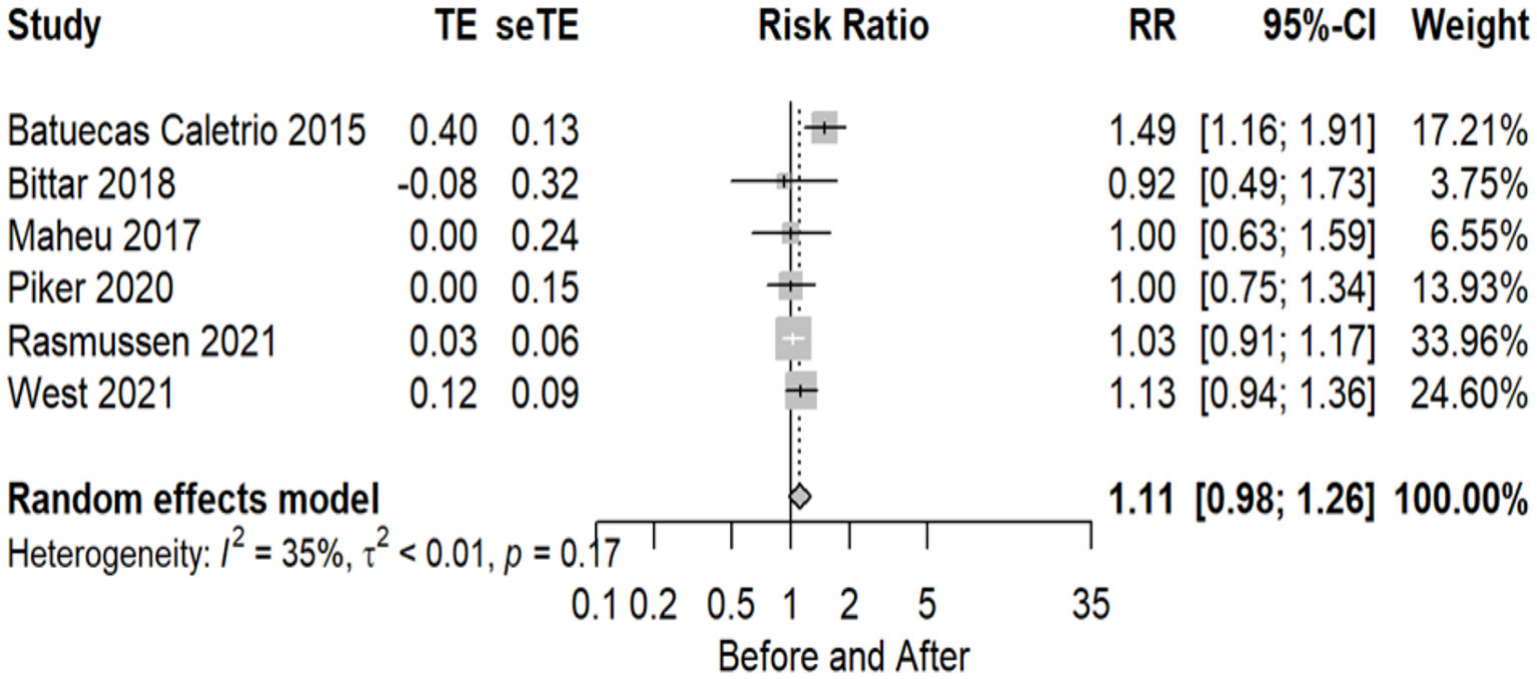
Forest plot (showing relative effect sizes) for the VHIT test.

The number of implant patients included in the meta-analysis who were evaluated using the VHIT test, with normal and abnormal test results before and after CI surgery, totaled 6 studies (20%), where in total, 151 individuals were evaluated as normal before and 121 as normal after implant. Statistical analysis revealed a non-significant effect of CI surgery on VHIT test results (RR = 1.11 95% CI = 0.98–1.26 *P* = 0.1025). There is no significant evidence of substantial variability in the results observed in these studies dues to the *P*-value, however, among all the tests, vHIT proved to be the least heterogeneous (I2 = 35%, *P* = 0.17). The forest plot indicating the relative strength of each study included in the meta-analysis is illustrated in Figure 5. The table containing all the articles that used the vHIT test for their analysis is found in Table 5.

**Table 5:** Number of subjects with normal and abnormal testing results before and after surgery in studies included in the meta-analysis for vHIT test.

**Study**	**Normal pre**	**Abnormal pre**	**Normal post**	**Abnormal post**	**Number pre**	**Number post**
Bittar et al. (39)	19	12	4	2	31	6
Batuecas-Caletrio et al. (24)	30	0	20	10	30	30
Maheu et al. (34)	4	0	4	0	4	4
Barbara et al. (26)	15	13	15	12	28	27
Piker et al. (35)	9	1	9	1	10	10
Rasmussen et al. (31)	40	3	39	4	43	43
West et al. (36)	34	3	30	7	37	37

### VNG test results

The number of implanted patients included in the meta-analysis who were evaluated using the VNG test, with normal and abnormal test results before and after CI surgery, totaled 6 studies (20%), where altogether 90 subjects were evaluated as normal before, and 68 as normal after implant. Statistical analysis revealed a non-significant effect of CI surgery on VNG test results (RR = 1.10 95% CI = 0.83–1.45 *P* = 0.4991). There is no significant evidence of substantial variability in the results observed in these studies (I2 = 50%, *P* = 0.07).

The VNG is a set of tests, which may include, among others, pendular tracking, rotating chair, postural, spontaneous, semi-spontaneous (the latter with eyes open or closed), and optokinetic, including caloric testing. Of the 6 studies that cited the use of VNG, 5 of them (24, 25, 29, 30, 53) described only the caloric test in their assessment methods, not describing the other tests used in the battery. Only one author (40) indicated, in addition to the caloric test, the static stabilometry and rotatory chair as the tests included in the VNG.

**Figure 6:**
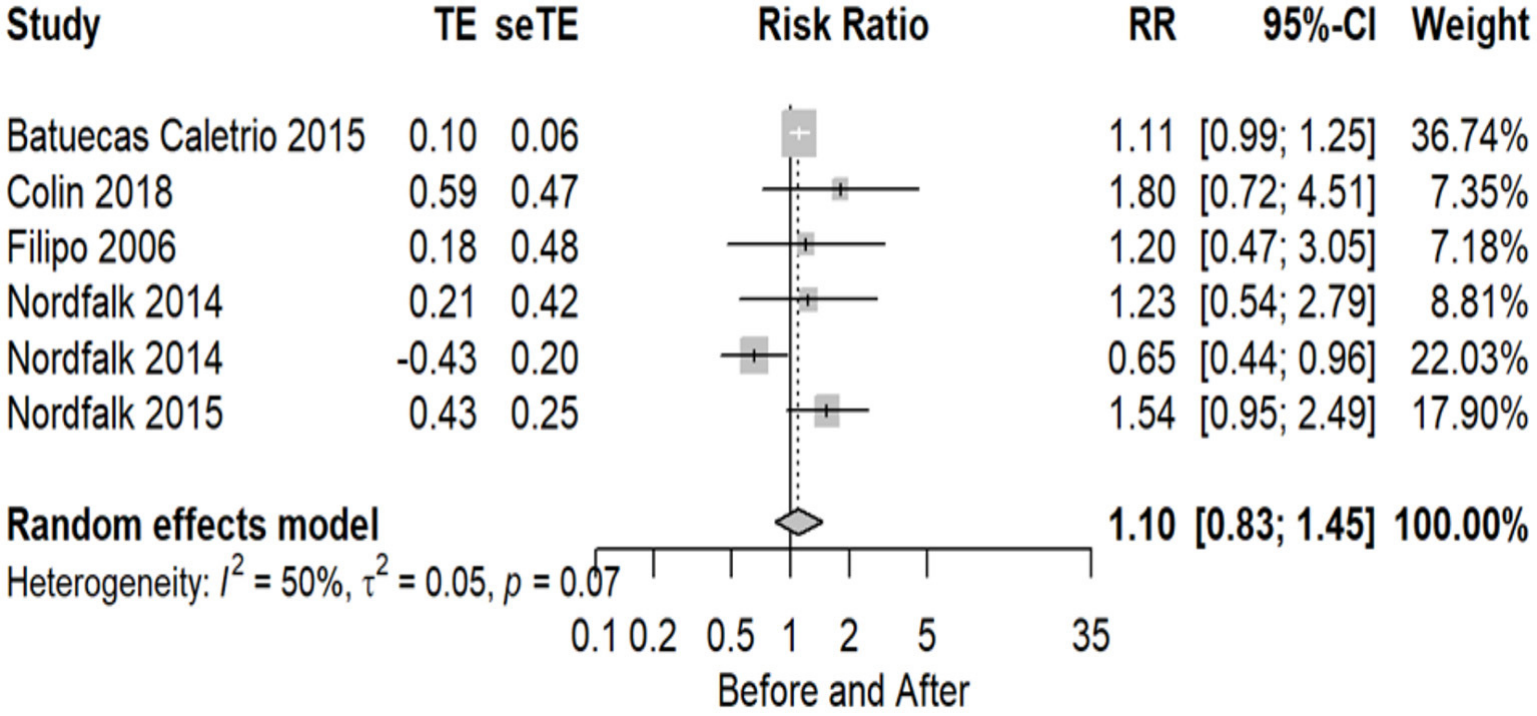
Forest plot (showing relative effect sizes) for the VNG test.

The forest plot indicating the relative strength of each study included in the meta-analysis is illustrated in Figure 6. The table containing all the articles that used the VNG test for their analysis is found in Table 6.

**Table 6:** Number of subjects with normal and abnormal testing results before and after surgery in studies included in the meta-analysis for VNG test.

**Study**	**Normal pre**	**Abnormal pre**	**Normal post**	**Abnormal post**	**Number pre**	**Number post**
Nordfalk et al. (29)	8	5	4	4	13	8
Nordfalk et al. (29)	17	15	14	3	32	17
Nordfalk et al. (30)	20	10	13	17	30	30
Batuecas-Caletrio et al. (24)	30	0	27	3	30	30
Colin et al. (25)	9	13	5	17	22	22
Filipo et al. (40)	6	8	5	9	14	14

Normal pre, number of people with normal test results before surgery; Abnormal pre, number of people with abnormal test results before surgery; Normal pós, number of people with normal test results after surgery; Abnormal pós, number of people with abnormal test results after surgery; Number pré, number of people tested before surgery; Number pós, number of people tested after surgery; RW, Round window; CCL, Cochleosto.

## Discussion

To identify evidence in the scientific literature and assess the interference of the different effects of CI placement on vestibular function in adult patients who underwent this procedure, we developed this meta-analysis by analyzing the results of studies with vestibular tests since there is no consensus in the literature on the subject. We included patients who underwent unilateral and bilateral cochlear implant and evaluated their vestibular function before and after surgery. We analyzed the results of studies related to the cochlear implant in several situations and performed a global analysis of the influence of the cochlear implant on vestibular function. It is important to note that 3 studies were performed by the same group (29, 30, 53). However, Nordfalk et al. have different sets of patient populations, so these studies did not have sample overlap.

For an adequate evaluation of possible residual symptoms, it is important to detail the otoneurological complaints (56) to identify patients who had complaints before the surgical procedure, those who improved, how many started having symptoms after the implant, and how many remained asymptomatic. It becomes necessary to evaluate before and after implant to help identify vestibular dysfunctions.

The importance of documenting vestibular function before CI surgery lies in three main aspects: knowledge of the functioning of stimuli and responses of both labyrinths, which guides the side to be implanted (13); the management of possible vestibular symptoms during the postoperative period (40); and, finally, the prognosis of this subject regarding body balance.

This meta-analysis showed great variability in the test results. This variability might be due to the different testing measures employed. Both HIT and caloric tests are strongly affected by the lateral semicircular canal function. VEMP testing is strongly influenced by the saccular function (57). Posturography testing is closely related to the compensatory mechanisms of postural performance (57). Each of the tests presented for the quantitative analysis examines a different structure of the vestibular system. Thus, it is not surprising that there is a discrepancy in the results of vestibular function tests in the comparison before and after surgery. For this reason, the literature highlights the importance of performing more than one vestibular function test before and after surgery. In a systematic review with meta-analysis, it was revealed that there is a variation in which of the five vestibular sensors (saccule, utricle, superior semicircular canal, horizontal and posterior) show changes after cochlear implant surgery, which may affect up to four of them. Therefore, a single test may not correlate with patients' symptoms, and the most efficient assessment is one that explores different vestibular sensors performing different vestibular tests (58).

Regarding the evaluation procedures, despite the differences in the parameters analyzed in each article, we standardized this review as to the types of tests applied to better evaluate symptom perception and observed that 19 studies used 1 of the vestibular function tests present in the inclusion criteria, 12 articles used 2 of the tests to evaluate cochlear implant recipients before and after surgery, and 7 performed their evaluations through 3 tests. It was observed that the studies selected for this meta-analysis did not present any study that used the VENG test. Thus, it is understood that there is a set of vestibular tests that are applied in different ways, where there is no standard stipulated test for the analysis of vestibular function in patients undergoing cochlear implants.

In view of this, based on the interpretation of the results of this set of tests, it appears that CI may affect some aspects of vestibular function (33). However, not all studies reported their criteria for performing them, and we must take into account that the variability may also be partially explained by differences in criteria and/or testing techniques, as well as by the cut-off point for determining whether test results are considered normal or abnormal (59). Although tests for evaluating vestibular function are well established in the literature, it was observed that there is still no single standard for such analyses. Several factors may be responsible for the variability among studies, such as the age range, the test settings, and the timing of the postoperative retest; of these, Kluenter's study (41) showed an important degree of confidence.

Another factor that contributes to the variability of the results is the fact that CI users are not a homogeneous population. In general they belong to different age groups, which can involve newborns to older adults suffering from severe to profound deafness. Thus, since age can affect vestibular function before, after, or before and after CI surgery (57), we chose to perform this meta-analysis by differentiating and restricting the sample only among adults over 18 years of age, minimizing age variation bias, a factor that showed high heterogeneity among studies on the subject, as evidenced in Ibrahim's meta-analysis (57); nevertheless, in our study, age variation still occurred, with a minimum of 18 years and a maximum of 86 years. Thus, since age can affect vestibular function before, after, or before and after CI surgery (57) would be desirable to perform an analysis on each group (adults or elderly people) or at least secondary analyses. However, we did not find enough separate data from older adults to perform such analyses.

The analysis where the studies were still heterogeneous occurred in the VEMP (I2 = 60%, *P* = 0.01), caloric test (I2 = 63%, *P* = 0.01), and HIT tests (I2 = 64%, *P* = 0.02); however, in the other tests, VNG (I2 = 50%, *P* = 0.07) and VHIT (I2 = 35%, *P* = 0.17), this could no longer be observed from the moment the division of groups between children and adults occurred, which already distinguishes the population in relation to the other tests.

From the results grouped in the current meta-analysis, when analyzing the values considered abnormal in the tests presented, it was found that before surgery, 46.4% had abnormal VEMP test results, 33.9% had abnormal caloric test results, 32.2% had abnormal HIT results, 17.4% in VHIT, and 36.1% had abnormal VNG test results, therefore, it is understood that within the patients who were evaluated before the surgical procedure, <50% had vestibular alteration before cochlear implant placement. However, although technically and numerically speaking, 46.4% are a minority, it is much higher than the general population, and, for that reason, it needs a careful look from the health professionals involved. When analyzing the outcomes found from the results about tests considered normal after surgery, 51.6% maintained normal caloric function after surgery, 59.8% maintained normal HIT results, 64% of patients had normal VHIT results, 56.1% also remained normal after surgery in the VNG test, and in the VEMP test, only 31.2% maintained normal results. Thus, it is observed that the impact of CI surgery on the vestibular apparatus was observed with clinically significant changes only in the VEMP test, and all other tests analyzed did not present significant symptoms after surgery.

The study by Melvin draws attention because it also showed no abnormality in the HIT test in any of the individuals evaluated, both before and after implant. In our study, only two studies (9, 33) had a relatively larger number of patients who maintained normal VEMP test results postoperatively. This may be due to the use of bone conduction VEMP, which is more sensitive compared to air conduction VEMP (9). It is worth noting that some conditions, such as ototoxic drug use or Ménière's disease, may be present in CI users and may limit the interpretation of abnormal balance tests if the test is done only postoperatively (57). However, for the most part, our studies did not report the detailed medical history of the patients to be conclusive.

We found that CI surgery can significantly affect the results of VEMP and caloric test. This finding is in agreement with the systematic review by Kuang et al. (60) who it showed that 37% of patients had reduced reflex and 34% had caloric asymmetry after CI surgery. The review published by Abouzayd et al. with the objective of determining the best test to assess vestibular function before and after CI surgery, reported that the caloric test is less sensitive, the VEMP results are mostly impaired, and the HIT results are usually preserved. In our analysis, the sensitivity found in the VEMP test also has a more meaningful significance in relation to the caloric test, both being the only sensitive tests to assess vestibular function in cochlear implants. In relation to the HIT test, we also did not find significance only an important heterogeneity (58).

Our meta-analysis found a similarity in the results of the pre-and post-surgical groups regarding the analysis of the HIT, VHIT, VNG, tests, where the three tests presented very small number of risks, without statistical significance between them, presenting only a difference of heterogeneity between groups. However, unlike all the previous tests, we found in the results of the VEMP test and caloric test, a significant difference when comparing pre-surgery with post-surgery. Thus, our study provides evidence that CI surgery can significantly affect some vestibular test results of VEMP and caloric test, the latter being of lower sensitivity compared to the former, and confirms that it is important to perform vestibular function assessments and follow a case-by-case approach with CI surgery candidates, based on each patient's history and symptoms (58).

Many authors believe that CI has a negative impact on vestibular function. However, not all implanted patients have postoperative complaints (11, 33, 41, 59); moreover, patients with CI report different symptoms after surgery. Bonutti further explains that cochlear implant surgery can affect the vestibular system, not only in the implanted ear but also in the non-implanted ear, with post-caloric nystagmus areflexia predominating. However, vestibular symptoms occur in a smaller proportion of affected individuals, and there may even be improvement in vestibular disturbances after cochlear implant surgery (19). In Imai, CI leads to a slight deterioration of utricular function, but not enough to cause vertigo (27). Jutila points out that subsequent high-frequency loss of vestibular function or onset of vestibular symptoms after cochlear implant surgery is rare but possible. This should be considered when counseling the patient, especially if bilateral cochlear implant surgery is being considered (38). It should also be noted that according to the study by Fina et al. (61), derived from a case–control study embedded within an ongoing cochlear implant cohort study, which served as hypothesis-generating data, individuals with a history of preimplantation dizziness, especially Ménière's disease, preimplantation abnormal computed dynamic posturography, older patients, and patients with a later age at onset of hearing loss were more likely to experience postoperative dizziness than those without a history of dizziness preoperatively, or in younger patients, or with earlier onsets.

It is important to highlight the limitations of vestibular testing in a surgical population and the limitations of this type of study design (i.e., pre-and postoperative vestibular tests) to reach a valid conclusion. For example, about cVEMP, this meta-analysis studies the results as normal or abnormal after implantation. Thus, it cannot yet be concluded that the statistical analysis revealed a significant detrimental effect of CI surgery on VEMP results because according to Merchant et al. (62), it is known that postoperative CI ears may have conductive hearing loss and airway-induced cVEMPs, which may not be a valid measure in this population. Another example, Patki et al. (63) showed in their study that much of the variance in the caloric test is due to mastoid air/bone, and we know that the mastoid can be altered after CI; as with air-induced cVEMPs, a caloric test cannot be interpreted directly after a CI. These studies need to be interpreted with great caution because a direct statistical analysis without further analysis is misleading and we cannot safely conclude that “statistical analysis revealed a significant detrimental effect of CI surgery on VEMP results” means that there was damage to the otolith.

We noted that cochlear implant surgery presents reports of vestibular disorders. This meta-analysis and the systematic review confirm with considerable variability and heterogeneity in test results, which may be due to differences in the design of each study, the characteristics of the research participants, and the different test measures employed. The type of surgery, pre-and post-surgical follow-up, and the procedures for assessing vestibular function during this process were also different. The heterogeneity and non-significance observed in the tests in this meta-analysis demonstrate that further studies should be performed to determine the improvement in standardized test scores after implant in this patient population.

The heterogeneity of study designs, the wide variety of the study population, and factors such as pre-existing symptoms that are not assessed as determining conditions are major barriers to study completion.

In summary, several factors may contribute to the variability of results within and between vestibular function tests before and after CI surgery, which are difficult to control. These factors include age and etiology of hearing loss, different cut-off points used for the evaluation of vestibular tests, the surgical technique used, and the incidence of inner ear trauma.

## Conclusion

The results of this systematic review with meta-analysis indicate statistically significant differences in the tests of vestibular function were detected using VEMP and caloric test in the comparison before and after cochlear implant placement surgery in adults and the elderly.

Comparing abnormal and normal results after implant surgery and analyzing the outcomes found from the results of tests considered normal after surgery, the vestibular apparatus introduces evaluated mostly as abnormal results only in the VEMP test. All other tests analyzed maintained a percentage of normal results after cochlear implant surgery. However, the potential effects of surgery on the vestibular system must be well evaluated by tests that investigate the vestibular function completely, before and after implantation, as well as discussed with CI candidates before surgery.

This meta-analysis and systematic review confirm considerable variability and heterogeneity in test results, which may be due to differences in the design of each study, the characteristics of the research participants, and the different test measures employed.

## Data availability statement

The original contributions presented in the study are included in the article/Supplementary material, further inquiries can be directed to the corresponding author/s.

## Author contributions

Conceptualization: FV, LP, WM, IS, JL, NS, NT-S, and FB. Data curation: FV, WM, and IS. Formal analysis: FV and WM. Investigation: JL, NS, and FV. Methodology: FV and LP. Project administration, resources, and supervision: FV and FB. Software: FV, WM, LP, and JL. Validation: FV, FB, and LP. Visualization: FV and NT-S. Writing–original draft: FV. Writing–review and editing: FV, FB, LP, and NT-S. All authors contributed to the article and approved the submitted version.

## Conflict of interest

The authors declare that the research was conducted in the absence of any commercial or financial relationships that could be construed as a potential conflict of interest.

## Publisher's note

All claims expressed in this article are solely those of the authors and do not necessarily represent those of their affiliated organizations, or those of the publisher, the editors and the reviewers. Any product that may be evaluated in this article, or claim that may be made by its manufacturer, is not guaranteed or endorsed by the publisher.
