# Supplementary material for: The effect of cochlear implant surgery on vestibular function in adults: A meta-analysis study
Source: Front Neurol. 2022 Aug 10;13:947589. doi: 10.3389/fneur.2022.947589 (PMC9402268; doi:10.3389/fneur.2022.947589)
Supplement: Supplementary file 1 [file Table_1.docx]

| Publishing | Comparison | SAMPLE | AGE | AGE (m e dp) | TRIALS | Surgery | PRE- AND POST-SYMPTOMS | implant | Study Outcome |
| --- | --- | --- | --- | --- | --- | --- | --- | --- | --- |
| Bujang et al. | IC Group and control | 20 | 21-43 years | GIC (28+-6.2 years) and control (28+-6.4 years) | Posturography | CCL | The control group and the group with CI had no symptoms pre- and post. | Unilateral | The results suggest that the surgery did not interfere with the function of the vestibular organs. |
| Parietti-  Winkler et al. | IC Group and control | 20 | GIC:  27 – 72  years  Control  24 – 71  years | GIC: 55 ± 20 control: 63 ± 16 years | Posturography, VNG | JR and CCL | No participants declared dizziness or vertigo. | Unilateral | The surgery did not change the vestibular status of the patients. |
| Barbara et al. | Pre and Post | 28 | 19-83 years | 57,4 | VEMP,  VHIT | JR | 7 patients had vertigo pre and 5 who did not have vertigo pre showed post, 3 patients kept their vertigo post and 4 did not keep their vertigo post | Unilateral (only 1 patient bilateral) | It revealed no significant change in vestibular function compared to the preoperative situation |
| Batuecas-Caletrio et al | Pre and Post | 30 | NI | 54 +- 10 | VHIT, VNG | JR e CCL | Patients who had vertigo pre-surgery were excluded, some patients had increased DHI after surgery. | Unilateral | The abnormalities identified in the VHIT in this study are clinically relevant (In three patients, changes in vHIT were observed (0.73, 0.74, 0.76), but the caloric response did not change. DHI scores also changed (average 0 before surgery to 16 after surgery)  because there is a relationship with subjective vestibular impairment in the DHI. |
| Bittar et al. | Pre and Post | 31 | NI | 49,6 ± 15,3 | VHIT | JR | 6 patients: dizziness;  2 patients: instability,  2 patients: unbalance and 2: vertigo after surgery | Unilateral | CI has little or no impact on vestibular function. |
| Black et al. | Pre and Post | 5 | 50-70  years | NI | P. calórica | NI | No symptoms of vertigo.  Spatial disorientation or nausea were caused on activation of their prostheses. | NI | Apparently, patients compensate very quickly for electrical stimuli unless the electrodes are in close proximity to the vestibular nerve. |
| Colin et al. | Pre and Post | 22 | 18-84 years | NI | VEMP, HIT, VNG | JR e CCL | 11 cases of vertigo before surgery; 11 cases of vertigo post and 15 reported no change in balance; 3 reported improvement and 4 reported worsening balance post. | NI | No significant correlations have emerged between symptoms and postoperative objective test results. Cochlear implant rarely appears to induce long-term vertigo. |
| Coordes et al. | Pre and Post | 140 | 20-73 years | Group 1: 60 Group 2: 50 | VEMP | NI | Patients (from Group 1) who reported  preoperative sound-induced vertigo were excluded from the study. 29 patients  confirmed the occurrence  of postoperative sound-induced vertigo. | NI | Our results show that sound-induced vertigo over environmental noise after cochlear implant is a frequent complaint in about one fifth of all cochlear implant patients |
| Enticott et al. | Pre and Post | 146 (86 took caloric test) | 37- 84 years | NI | Caloric Test | CCL | Most subjects in the preoperative period did not have  dizziness or vestibular symptoms; 33% of subjects 70 years and older reported symptoms after surgery. | Bilateral (1) and unilateral | Overall, about one third of implant patients experienced significant vestibular disturbance lasting 1 week or more after surgery. |
| Ernst et al. | Pre and Post | 54 | 18-62 years | 43 | VEMP | CCL | The subjective complaints of the patients, which remained unchanged for at least 6 months postoperatively, were typically of "slipping" and "falling" sensations. | NI | The postoperative loss of VEMP in most of the patients  most of the patients implanted in this study probably occurred due to surgical trauma |
| Filipo et al. | Pre and Post | G1-21  G2-72  T: 93 | G1:  18-79  G2:  17-51 | Group 1: 41; Group 2: 51 | VNG | CCL | 19 experienced vertigo before surgery; 25 reported vertigo right after surgery 10 continued to have the same symptoms just before activation of the CI. 1 patient reported accentuation of vertigo after surgery, while 6 of them experienced a first episode of vertigo. | Unilateral and Bilateral | It is possible to state that the initial negative impact exerted by CI on the vestibular system seems to be less dramatic when considering the outcome of complex compensatory phenomena. |
| Frodlund et al | Pre and Post | 43 | G1:38-83  G2:28-85  G3:37-86 | Group 1:58,6; Group 2: 64  Group 3: 62,2 | VNG | JR | 7 reported vertigo; and some patients reported dizziness after surgery. | NI | There was no significant post-surgical change in vestibular function in any of the three groups evaluated |
| Imai et al. | Pre and Post | 12 | 7-82 years * | 66 | VEMP | JR | 2 patients had symptoms pre and post, and 2 patients had symptoms only post. | Unilateral and one bilateral | The results indicate that CI causes a small deterioration of utricle function that is insufficient to result in vertigo or deterioration of lVOR. |
| Jutila et al | Pre and Post | 44 | 30-76 years | 55 | HIT | CCL and 1 JR | The dizziness prevalence and intensity score was 1.4 +- 0.5 preoperatively, 1.4 +- 0.7 early postoperatively, and 1.3 +- 0.5 late postoperatively.  operative period and 1.3 +- 0.5 in the late postoperative period. | Unilateral | Thus, changing the horizontal VOR in the  high-frequency range seems to be a possible, but rare  complication after CI. |
| Kiyomizu et al. | Pre and Post | 23 | 36-75 years | NI | Caloric test | NI | 7 patients had some type of subjective dizziness after surgery. Of which, 4 cases had recurrent dizziness after surgery, and 3 cases had transient dizziness after  surgery. 3  had paroxysmal vertigo after surgery. | NI | Cases with deterioration in caloric response after surgery had a significant risk of paroxysmal vertigo after surgery. |
| Kluenter et al. | IC group and control | IC-24   Control-19 | IC  20 – 78 years Control 20 - 58 years | 59 – IC 49 - Controle | Caloric test and posturography | CCL | The patients did not complain of dizziness or other signs of impaired balance function. | Unilateral | Significant vibratory disturbance of the semicircular canals or otoliths does not seem to have occurred in the patients, since all but one patient showed no deterioration of caloric function on the operated side. |
| Louza et al. | Pre and Post | 30 | NI | 56 ± 19 | VEMP,  Caloric | JR | 15 patients had vertigo symptoms after implant, in the preoperative period no patient had vestibular symptoms | Unilateral | Variability in insertion depth had no influence on the development of vertigo symptoms.  Regarding the objective vestibular tests, no direct correlation  with vertigo symptoms was found. |
| Maheu et al. | IC group and control | 17 | 34-49 years | NI | vHIT, VEMP and posturography | NI | NI. | NI | If participants receive their implant in the  ear with worse vestibular function, the differences observed in postural control will be less significant.  the observed differences in postural control  after cochlear implant surgery. |
| Migliaccio et al. | Pre and Post | 16 | 27-65 years | 46 | HIT | CCL | 6 patients with vertigo | NI | Cochlear implant rarely causes a decline in vestibular function. Only one patient out of 11 (9%) experienced a decline in vestibular function in the implanted ear. |
| Miwa et al. | Pre and Post | 9 | 35-79 years | 56± 22 | Cloric test, VEMP and posturography | CCL | 1 patient with vertigo, 4 buzzing sounds and 1 dizziness after surgery. | NI | Postoperative caloric and VEMP  in CI-off mode exhibited deterioration relative to  the preoperative responses |
| Melvin et al. | Pre and Post | 28 | 23-69 years | 46 | HIT, VEMP, Caloric test | CCL | One patient had severe postoperative vertigo | 30 unilateral CI, 3 with previous CI underwent the 2nd, 1 bilateral | The incidence of vestibular damage during unilateral CI was low |
| Nordfalk et al. | Pre and Post | 13 | 32-61 years | NI | VNG, VEMP | 12 CCL e 1 JR | 4 patients had dizziness | Unilateral | Positive cVEMP test responses before surgery were  observed in 9 of the 12 patients tested. 5 had an  intact positive cVEMP response after surgery. |
| Nordfalk et al. | Pre and Post | 39 | 18-85 years | 57,5±17,2 | VEMP, VNG | JR | There were new symptoms of vertigo: 1 patient in the +FLEX28 group and 2 patients in the + Flex solft group | Unilateral | Fourteen individuals in total have an intact VEMP response after surgery. |
| Oikawa et al. | Grupo com IC e controle | 16 | 20-61 years | 44 | Posturography | NI | No patient reported dizziness | Unilateral | The total length and the total sway area with eyes open were not different  between patients with CI and subjects without CI. This result suggests that asymmetric vestibular function was well compensated in the static condition, CI activation has beneficial effect on body balance function. |
| Shute et al. | Pre and Post | 37 | 20-84 years | 55 | vHIT | NI | 8 patients had vertigo, 4 had imbalance and 13 had dizziness after surgery | NI | Imbalance is common in the first 30 days after CI, occurring in  35% of subjects. Despite this, hSCC function  (as measured by vHIT) was preserved. |
| Vibert et al. | Pre and Post | 12 | 9-77 years * | 48 | Caloric test | CCL | One patient had dizziness and one had vertigo | NI | Initial ENG demonstrated that CI disrupts lateral semicircular canal function in: 20% of cases. However, after several weeks, canal function returned to  returned to normal numbers |
| Nordfalk et al | Pre and Post | G1-17  G2-17  T: 34 | G1:  35-85  G2:  26-69 years | NI | VEMP, VNG | JR and CCL | 5 patients in the CCL group and 2 in the JL group had a new symptom of dizziness afterwards. | Unilateral | There was no significant difference between the groups regarding change in vestibular function. |
| Wiszomirskaa et al. | Pre and Post | 21 | NI | 50.66 ± 18.02 | Posturography | JR | NI | Unilateral | The analysis of pre- and post- implant results revealed no significant changes, except for improvement in the mid-lateral stability index.  The results of the fall risk test improved, which allows us to assume that the implant pattern through the round window membrane has produced the expected results. |
| Stieger et al. | Pre and Post | 30 | NI | 59± 15.4 | Posturography | NI | NI | Unilateral | Significant worsening of balance during walking for those under 60 years of age and those without balance pathology preoperatively. Those who would be considered  prone to have balance problems (those older than 60 years and those with preoperative  and those with preoperative balance deficits) had no significant worsening in balance control. |
| Brey et al. | Pre and Post | 52 | 17-77 years | 56,5 | Caloric test and Posturography | NI | 5 patients onset of vestibular deficit post | Unilateral | The risk of inducing peripheral weakness in the implanted ear was 41% if the pre vestibular function was normal. In the under-60 age group, patients did not appear to have persistent vestibular complications. In the age group above 60 years, there was a significant drop in the caloric response of the implanted ear. |
| Meli et al. | Pre and Post | 25 | 24-75 years | 45,3 | HIT, VEMP, Posturography (static stabilometry) | JR | 3 patients dizziness  1 spontaneous nystagmus | 6 patients received bilateral CI; 5 patients received sequential CI and 14 patients received a monaural CI. | Patients with symptoms had a  greater response on the caloric test before surgery. Patients with CI showed good compensation, suggesting that the impact on the vestibular system should not be considered a negative effect  of cochlear implant. |
| ITO | Pre and Post | 55 | 18 + | NI | Caloric test | NI | 15 patients had early dizziness, 9 had prolonged dizziness, and 2 patients had the delayed type. 2 patients felt dizziness when they wore the implant, the other 9 reported no relationship between wearing the  implant and dizziness. | NI | The results indicate that cochlear implant did not strongly affect vestibular function, or that vestibular function was compensated very quickly. |
| Guan et al. | Pre and Post | 15 | 18-70 | 37.46 ± 15.32 | VEMP, vHIT, Caloric test | JR | The results of the questionnaires on vertigo were not significantly different between the periods. | Unilateral | The abnormal rates in caloric and VEMP tests increased greatly  from pre- to post- implant indicating the negative effect of CI on vestibular functions. The vHIT  test showed no significant difference in abnormal rates between  pre- and post- implant, suggesting that the otolith  and low frequency SCC are more vulnerable. |
| Rasmussen et al. | Pre and Post | 35 | 26–85 | 59 | VEMP, vHIT, Caloric test | JR | Despite vestibular dysfunction, a large proportion of patients report fewer or unchanged vestibular symptoms. | 17 left, 16 right, 2 bilateral | Implant can worsen vestibular function and long-term effects tend to stabilize rather than deteriorate vestibular function. vHIT, caloric irrigation, and cVEMP all measured an overall worsening of vestibular function  at long-term postoperative follow-up. |
| Piker et al. | Pre and Post | 16 | 26-85 | 62 | vHIT, Caloric test | JR | No patient showed  evidence of vestibular dysfunction before surgery and 7/10 patients  reported a DHI score of 0 (no dizziness disability). After surgery 3 patients reported dizziness. | Unilateral | The cases illustrate that group average differences often miss significant findings in individual patients and how, in isolation, a single vestibular test cannot provide adequate information about the entire  system. |
| Tsukada and Usami | Pre and Post | 66 | 7 -70* | 46.6 ± 18.3 | Caloric test and VEMP | JR and CCL | 13 patients had symptoms before surgery, after surgery 4 patients who did not present symptoms began to have symptoms. | Unilateral | The results showed relatively better preservation  of postoperative vestibular functions compared to previous reports. When considering factors related to  vestibular preservation, there were no significant differences between vestibular preservation for age at implant  side, gender.  Thus, one of the reasons for these better results is probably  the surgical technique applied, such as JR and the use of  flexible electrodes. |
| Bernard-Demanze et al. | Group with IC and control | IC-16  control - 13 | NI | IC- 59.7 +-12.3  control: 39.5 +- 9.1 | Posturography | NI | 3 patients reported vertigo and instability: In the dark (3), light (3) head movement (3), prosthesis off (2), supermarket (3) | Unilateral | The study clearly shows that the postural performance of CI patients is inferior compared to controls in both  static and dynamic conditions, especially without vision.  We conclude, that patients with long-term (> 1 year) CI (1)  have impaired postural performance, and (2) exhibit a vision-based replacement strategy sensor |
| West et al. | Pre and Post | 40 | 26-86 | NI | VEMP e vHIT | JR | 20 patients reported dizzines post surgery | Unilateral | The results show that vestibular function is  affected in various ways, depending on the outcome  considered. The results suggest a high rate  of vestibular loss after cochlear implant, which is  only partly associated with self-reported symptoms. |
